# Characteristics of urine spraying and scraping the ground with hind paws as scent-marking of captive cheetahs (*Acinonyx jubatus*)

**DOI:** 10.1038/s41598-022-19257-7

**Published:** 2022-09-16

**Authors:** Kodzue Kinoshita, Misa Suzuki, Yuuta Sasaki, Aya Yonezawa, Hisayoshi Kamitani, Ryuta Okuda, Tatsuya Ishikawa, Kenta Tsukui, Shiro Kohshima

**Affiliations:** 1 Wildlife Research Center, Kyoto University, Tanaka-Sekiden 2-24, Sakyo, Kyoto, 606-8203 Japan; 2 Tama Zoological Park, Hino, Tokyo 191-0042 Japan; 3 Himeji Central Park, Himeji, Hyogo 679-2121 Japan; 4 Fuji Safari Park, Susono, Shizuoka 410-1231 Japan; 5 Adventure World (AWS Co. Ltd.), Nishimuro, Wakayama 649-2201 Japan

**Keywords:** Animal behaviour, Behavioural ecology

## Abstract

Olfactory communication is common in felids. We observed two scent-markings, urine spraying and scraping the ground with hind paws during excretion, of 25 captive cheetahs. We analyzed the association of sniffing with the timing of urine spraying and scraping, and differences in these behaviors based on sex, age, and captive environment to understand the olfactory communication among cheetahs. Both scent-markings were strongly associated with sniffing, especially scraping, and the presence or absence of scent was thought to be a trigger. Both behaviors were observed only in adults; scraping was observed only in males. To our knowledge, this study was first to confirm the discharge of secretions from the anal glands during scraping. The frequencies of both behaviors were significantly higher in males kept in shared enclosures containing other individuals than in males kept in monopolized enclosures, while there was no difference in the frequencies among females. Female cheetahs are solitary and have non-exclusive home range, whereas male cheetahs are either solitary or live in coalition groups and there are territorial and non-territorial males. Our results could be attributed to the differences in sociality between the sexes and effect of the living environment.

## Introduction

Mammals communicate in various ways, including olfaction, hearing, and vision. Many felids that are widely distributed in diverse environments on the Earth, except Antarctica, Oceania, Madagascar, and Greenland, are solitarily or live in groups comprising several relatives. Given their diverse habitats and social structures species-specific ways of communication may exist. Many felids communicate with each other through cues such as vocalization and scent marking^1–5^. Such communication approach among felids is likely used to avoid unnecessary conflicts within and between species by marking territory and transmitting information among each other to increase breeding opportunities^3,5,6^.

Olfactory communication is thought to be the most common means of feline communication and has been reported in a variety of felids, including tigers (*Panthera tigris*^6^), servals (*Leptailurus serval*^7^), domestic cats (*Felis catus*^8^), black-footed cats (*Felis nigripes*^5^), snow leopards (*Panthera uncia*^9^), the Sunda clouded leopards (*Neofelis diardi*^10^), and small felids^2^. Generally, via olfaction, cheetahs convey information, including that on home ranges^3^, to other individuals through scents derived from feces, urine, sweat, and sebaceous gland secretions^11^ as well as by rubbing the body against trees and stones. Moreover, claw raking hat involves ground and tree scratching is also regarded a scent-marking as it leaves behind secretions from the interdigital and paw sweat glands, in addition to providing visual communication cues^3,12^.

The cheetah, *Acinonyx jubatus,* is classified as a vulnerable species by the International Union for Conservation of Nature (IUCN) because its numbers have declined in recent years owing to the reduction in its habitat and food resources caused by environmental deterioration and poaching^13^. Cheetahs possess unique social characteristics among felids. Unlike other felids where both the males and females are highly solitary, female cheetahs are solitary, whereas male cheetahs are either solitary or live in coalition groups usually consisting of littermates^14,15^. In addition, solitary males and males in coalitions roam either in small exclusive territories or in large home ranges overlapping with other male home ranges and territories, whereas females roam in large home ranges overlapping with those of other females and males^15,16^. Their behaviors of “urinating on a surface by backing up to a tree and pointing their penis backward to urinate horizontally or 60° upwards” and “urinating close to the ground in a semi-squatting position while repeatedly raking the ground with their hind feet around and over their urine” in the wild are thought to be scent-marking^17^. However, detailed quantitative analyses of scent-markings, such as their association with sniffing and the differences between excretion for scent marking and general excretion, are scarce. Therefore, to examine the scent-markings and their association with sniffing, we targeted captive animals as these can be observed comprehensively and quantitative data on their behavior can be easily collected. In addition, the scent-markings, were analyzed to determine the role of each of these behaviors in the cheetahs’ olfactory communication as well as the effect of location, sex, age, and captive environment on these behaviors.

## Methods

### Study animals

We monitored 25 cheetahs in four zoos (Zoos A, B, C, and D): 5 adult females, 14 adult males, 3 sub-adult females, and 3 sub-adult males. This study was conducted from October 2018 to November 2019, but the observation period and frequency varied for each individual (see Supplementary Table a). According to the findings of Eaton^18^ and Wielebnowski and Brown^19^, cheetahs aged 1 − 2 years and ≥ 2 years were treated as sub-adults and adults in this study, respectively. One female and two males that turned 2 years old during the study period were treated as adults. Information on each study animal is presented in Table 1.

**Table 1:** Information of study animals.

Individual	Breeding history (Yes: ✓)	Age at the time of observation	Zoo	Number of days observed
Female 1		1–2	C	10
Female 2		1	C	5
Female 3		1	C	5
Female 4		2	C	5
Female 5		4	A	10
Female 6	**✓**	6–7	C	10
Female 7		7	B	7
Female 8		10	A	10
Male 1		1	A	12
Male 2		1–2	C	12
Male 3		1–2	C	12
Male 4		3–4	D	5
Male 5		4	B	8
Male 6		5–6	D	5
Male 7		6–7	C	10
Male 8		6–7	C	10
Male 9	**✓**	8–9	C	12
Male 10	**✓**	8–9	C	12
Male 11		9	C	7
Male 12	**✓**	9–10	C	12
Male 13	**✓**	10	A	8
Male 14	**✓**	11	D(C)^a^	3 (2)^a^
Male 15		11	D(C)^a^	3 (2)^a^
Male 16		12	B	10
Male 17		13	A	18

^a^Moved on July 17, 2019.

### Scent conditions at the observation location

The cheetahs released into the outdoor enclosures during the daytime (9:30–17:00) were observed. The time and duration of release varied among individuals. At night, all observed cheetahs were housed in individual indoor facilities in separate male and female animal sheds. The conditions for scent marking and observation sites differed between the zoos.

### Observation in the monopolized enclosure: Zoos A and B

A monopolized enclosure was an outdoor enclosure where only one specific individual was released at all times of the day every day; the scents of other individuals were relatively milder in this enclosure than the scents in the shared enclosures. Sometimes different individual used the enclosure; therefore, it was not completely devoid of the scents of other individuals.

Three males (1, 13, and 17; Table 1) were studied to investigate the frequency of urine spraying. The 13-year-old (Male 17) and 1-year-old (Male 1) males, both previously exclusively released, were sequentially and alternately released into the paddock normally used by Male 13 (10-year-old) for approximately 25 min each, followed by the release of Male 13 into the same paddock. Male 17 had previously been intimidated by Male 13, indicating a dominant/submissive relationship between the two cheetahs (as stated by Yonezawa and Kamitani, a veterinarian and a keeper in the zoo A, respectively).

### Observation in the shared enclosure: Zoos C and D

In this outdoor enclosure, where multiple animals were released sequentially at different times or simultaneously on the same day, the individuals were released randomly one after another, depending on the daily schedule. Therefore, the scents of other individuals were considered to be stronger in this enclosure than those in the monopolized enclosure.

In Zoo C, although the individuals were housed separately in an indoor facility at night, two sets of 3 male siblings (Males 7, 8, 9 and Males 10, 14, 15), one set of 3 female siblings (Females 1, 2, 3), or one mother and two cubs were housed together at not only day-time but also night time. The behavior of the one mother and two cubs were not observed.

### Behavioral observation methods

All behaviors were recorded continuously when the cheetahs were released in the outdoor enclosure. One or two video cameras were set up on the fence to record the behaviors. In Zoo C, observations were also made directly from the visitor passage, because the longest distance from the passage to the animals was approximately 54 m. The time that the animals were released into the outdoor enclosure varied from 25 min to 6 h. To handle all data uniformly, the behavioral observations were carried out during the 25 min immediately after the animals were released to their outdoor enclosures. Some days, when the behavior could not be recorded because the animals were not within the camera range, were excluded from the analysis. The observation zoo and number of days of observations for each individual are shown in Table 1.

### Definition of behavior

**Figure 1:**
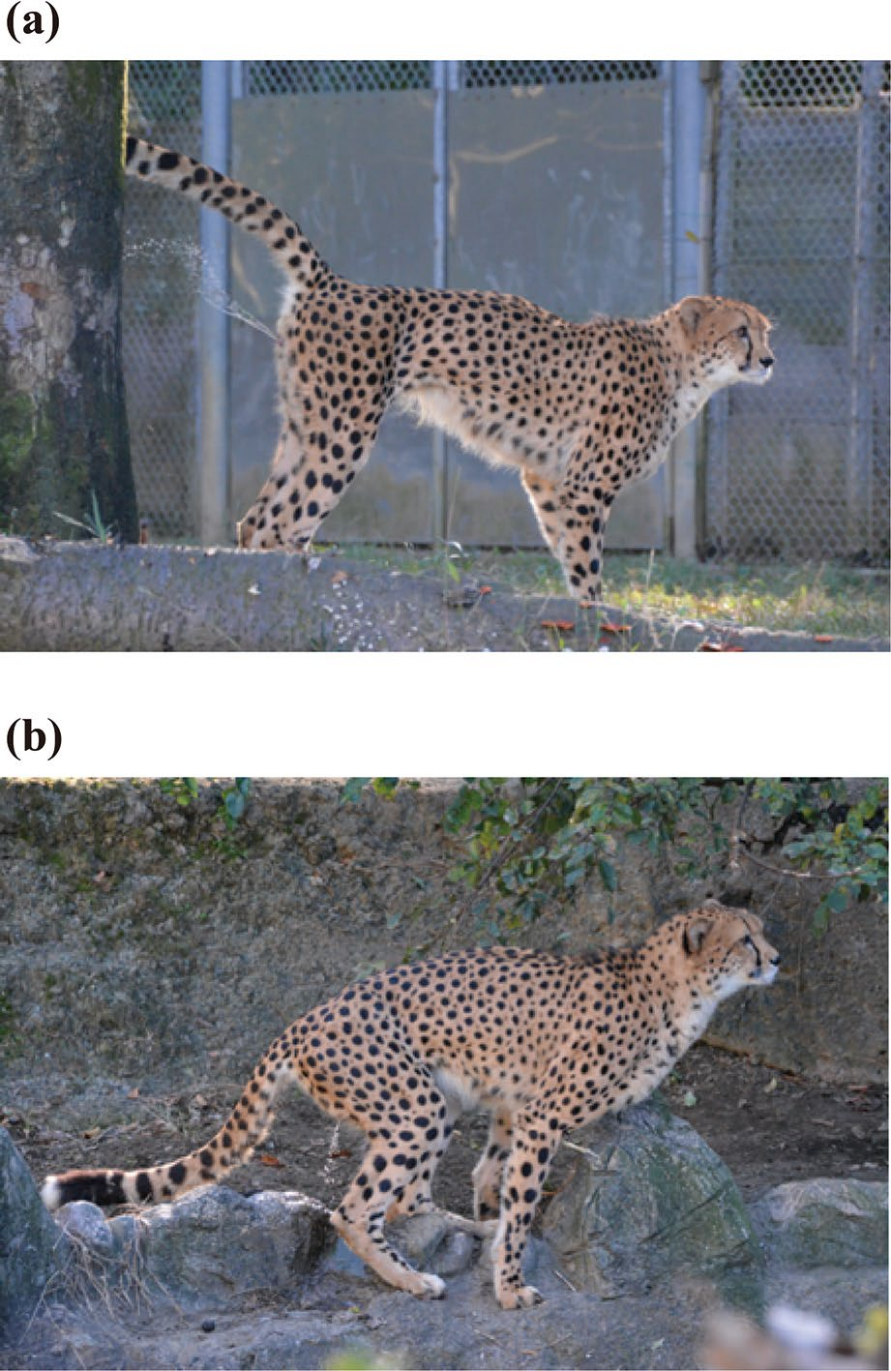
Behaviors of captive cheetahs that were thought to be scent-marking: (**a**) excretory posture at the time of urine spraying and (**b**) excretory posture at the time of scraping.

The scent-markings of urinating on the object with the tail raised above the hip was termed as *urine spraying* (Fig. 1a) and of bending the hindlimbs to lower the hips and urinating or defecating without raising the tail, and scraping the ground with their hind paws was termed as *scraping* (Fig. 1b). And urination and defecation without scraping were regarded as *other excretion*. During each behavior, we visually observed the elements being excreted. Positioning the nose within a distance of 5 cm from the target object for at least 1 s was regarded as *sniffing*. In addition to the frequency of these behaviors, the locations at which the behaviors were displayed, that is, the object sprayed with urine or the object against which cheetahs scraped, were recorded: wall or fence (at least 200-cm high), standing tree (at least 200-cm tall), stump (approximately 170 cm), rock (approximately 100 cm), stone (10–60 cm), fallen tree (10–50 cm), straw pile (approximately 3 cm), or the ground without nearby objects. In the case of scraping, the wall, fence, or standing tree were recorded when cheetahs scraped the hindlimbs were scraped near it. In the enclosures of Zoo A, the floor was concrete and did not have stumps, rocks, fallen trees, or straw piles. Zoo C had comparatively many objects including at least four low and horizontal objects (straw piles, fallen tree, stones, and rocks). Zoos B and D had at least one low and horizonal objects. Therefore, the frequency of each behavior on each target object was analyzed using only the data that we obtained from the enclosure where the target object was present.

### Statistical methods

The rate of each behavior was tested using binomial test or χ^2^ test, and differences owing to sex and captive environment, i.e., monopolized or shared enclosure, were examined using the Mann–Whitney *U* test. All tests were performed using SPSS version 23 (IBM Japan Ltd.). Statistical significance was set at *p* < 0.05.

### Compliance with ethical standards

This study was merely observational and was non-invasive; data were collected during zoo opening hours. Observations were made from the visitor passage as well as by recording behaviors using video cameras; therefore, the cheetahs experienced no additional disturbance.

### Ethical approval

The management of the captive cheetahs in this study followed the Code of Ethics of the Japanese Association of Zoos and Aquariums. Observation procedures were noninvasive, approved by each zoo, and were carried out according to the guidelines for animal studies in the wild and ethics in animal research issued by the Wildlife Research Center of Kyoto University.

## Results

Twenty-five animals (8 females and 17 males) were observed for 25 min each, resulting in a total observation time of 5250 min (1575 min for females and 3675 min for males). Urine spraying was observed 35 times in 3 adult females and 610 times in 13 adult males (645 times), and the frequency ranges were 0.01–0.11 and 0.02–0.39 times/min, respectively, indicating a significantly higher frequency in males (Mann–Whitney *U* test, *p* < 0.05) (Table 2 and Supplementary Table a). Scraping was observed 176 times in 10 adult males only, and the frequency range was 0.003–0.20 times/min (Table 2 and Supplementary Table b). Other excretion was observed 10 times (urination: 1 sub-adult male and 1 adult male; defecation: 3 times in 2 sub-adult females, 4 times in 3 adult males, 1 time in 1 adult female) (Supplementary Table c, Online Resource 1; Other excretion besides urine spraying and scraping).

**Table 2:** Frequency of urine spraying and scraping using hind paws observed in captive cheetahs for 25 min after release into enclosures.

Individual	Breeding history (Yes: ✓)	Age at the time of observation	Zoo	Urine spraying (times/min)	Scraping (times/min)
Female 1		1–2	C	0	0
Female 2		1	C	0	0
Female 3		1	C	0	0
Female 4		2	C	0	0
Female 5		4	A	0	0
Female 6	**✓**	6–7	C	0.04	0
Female 7		7	B	0.11	0
Female 8		10	A	0.01	0
Male 1		1	A	0	0
Male 2		1–2	C	0	0
Male 3		1–2	C	0	0
Male 4		3–4	D	0.21	0.02
Male 5		4	B	0.02	0
Male 6		5–6	D	0.29	0.01
Male 7		6–7	C	0.32	0.11
Male 8		6–7	C	0.24	0.16
Male 9	**✓**	8–9	C	0.24	0.04
Male 10	**✓**	8–9	C	0.39	0.09
Male 11		9	C	0	0
Male 12	**✓**	9–10	C	0.28	0.20
Male 13	**✓**	10	A	0.25	0
Male 14	**✓**	11	D(C)^a^	0.33	0.13
Male 15		11	D(C)^a^	0.17	0.11
Male 16		12	B	0.11	0.003
Male 17		13	A	0.22	0

^a^Moved on July 17, 2019.

### Characteristics of scraping: excretion and timing of scraping

**Figure 2:**
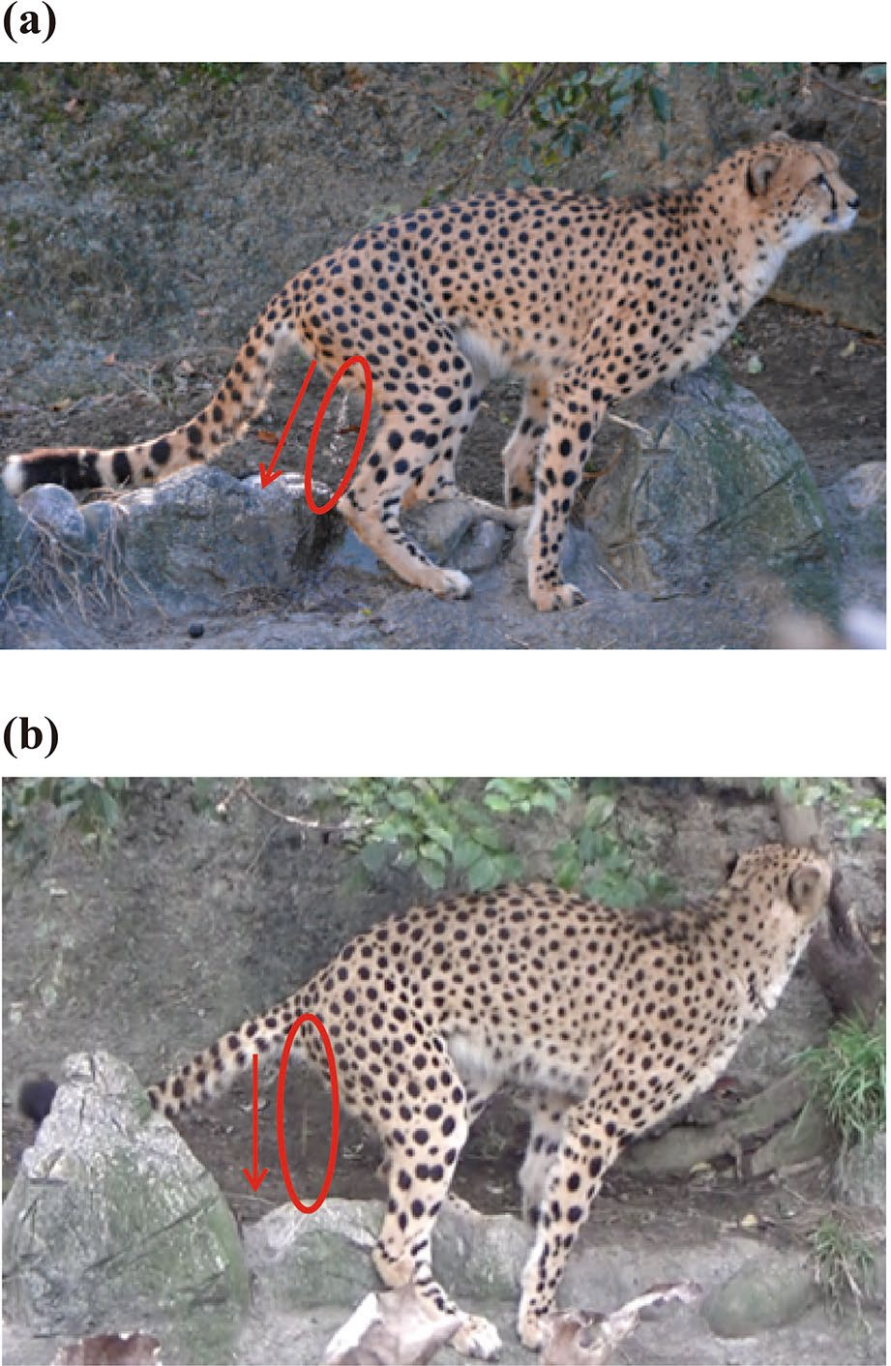
Comparison of postures when excreting urine and secretion during scraping behavior in captive cheetahs: (**a**) urination and (**b**) secretion discharge. The red arrows indicate the direction of release of the urine or secretion.

During the process of scraping, the excrement was visually confirmed in 101 (57.4%) out of the 176 observations (Table 3). There were 63 (62.4%) instances of urination and 44 (43.6%) instances of defecation. In addition to urination and defecation, brown or transparent secretions were observed 17 times (16.8%). Urine was released diagonally backward from the urethra during urination (Fig. 2a), whereas the secretions occurred from the vicinity of the anus almost perpendicular to the ground (Fig. 2b). When secreting, only the base of the tail was raised, and the tip of the tail swiftly moved up and down (Online Resource 2; Posture when excreting secretion during scraping). Therefore, the excretion of secretions differed from urination with respect to the excretion port and release angle. Furthermore, as shown in Table 4, brown or clear secretions were often excreted along with urine and feces (14 times, 82.4%); however, they were occasionally excreted without urine or feces (3 times, 16%). Therefore, this secretion was not regarded as part of the urine and feces.

**Table 3:** Excrement production during scraping and the timing of hind paws scraping.

	Times	%
1. Total number of observations	176	
2. The number of times the excrement was confirmed	101	57.4 (101/176)
** 2. 1. With urination**^**a**^	63	62.4 (63/101)
[The timing of hind paws scraping] Before excretion	4	6.3 (4/63)
[The timing of hind paws scraping] During and after excretion	59	93.7 (59/63)
** 2. 2. With defecation**	44	43.6 (44/101)
[The timing of hind paws scraping] Before excretion	19	43.2 (19/44)
[The timing of hind paws scraping] During and after excretion	25	56.8 (25/44)
** 2. 3. With brown or transparent secretions**	17	16.8 (17/101)
[The timing of hind paws scraping] Before excretion	5	29.4 (5/17)
[The timing of hind paws scraping] During and after excretion	12	70.6 (12/17)

^a^Indicates significant difference between “before excretion” and “during and after excretion” (*p* < 0.05: binomial test).

**Table 4:** Details of all instances of discharge of brown or clear secretion during the observation period seen in captive cheetahs.

Individual	Episode order					
	1	2	3	4	5	6
Male 7	Feces → Secretion					
Male 7	Feces → Secretion^br^					
Male 7	Urine → Feces	Feces → Secretion				
Male 8	Urine	Urine	Urine → Secretion			
Male 8	Urine	Feces	Feces → Secretion			
Male 12	Urine	Feces → Secretion	Urine → Secretion			
Male 12	Urine	Feces → Secretion	Urine			
Male 12	Urine	Feces → Secretion	Urine			
Male 12	Urine	Urine	Urine	Feces → Secretion^br^	Secretion	
Male 12	Urine	Feces	Secretion	Secretion	Urine	
Male 12	Urine	Urine	Urine	Urine	Feces → Secretion^br^ → Secretion	Urine
Male 12	Feces → Urine → Feces → Secretion^br^					
Male 14	Urine → Feces → Secretion^br^					
Male 15	Urine	Feces → Secretion^br^	Feces			

Brown secretion is indicated as Secretion^br^ and clear secretion as Secretion.

As shown in Table 3, scraping was mainly observed during and after excretion. In the 63 occasions of urination, the cheetahs often scraped during and after excretion (59 times, 93.7%) but not before excretion (4 times, 6.3%) (binomial test, *p* < 0.05). In the 44 occasions of defecation and 17 occasions of secretions, the cheetahs scraped during and after the processes: 25 times (56.8%) for feces (binomial test, *p* = 0.451), 12 times (70.6%) for secretions (binomial test, *p* = 0.143); there was no significant difference between the numbers of scraping during/after and scraping before excretion events. In all cases of excrement of urine, feces, and secretions, the hindlimbs were never rubbed only after excretion.

### Relationship of scent-marking with sniffing

In the observed 645 urine spraying instances and 176 scraping instances, the presence or absence of sniffing immediately before or after was observed in 558 urine spraying events (86.5%) and 164 scraping events (93.2%; Table 5). Of these, sniffing only immediately before excretion was observed on 512 occasions (91.8%) of urine spraying and 84 occasions (51.2%) of scraping (χ^2^ test, *p* < 0.05); sniffing only immediately after excretion was observed on no occasion of urine spraying and 8 occasions of scraping (4.9%); and sniffing both immediately before and after excretion was observed on 3 occasions of urine spraying (0.5%) and 63 occasions of scraping (38.4%) (χ^2^ test, *p* < 0.05). There was significant difference between the numbers of the instances of sniffing only before scraping and scraping without sniffing (χ^2^ test, *p* < 0.05). Among 7 occasions of other excretion which the presence or absence of sniffing was observed, 3 (42.9%) did not entail sniffing; in the remaining 4 occasions, sniffing was observed immediately before excretion (57.1%).

**Table 5:** Sniffing associated with urine spraying and scraping.

	Urine spraying		Scraping		Other excretion	
	Times	%	Times	%	Times	%
1. Total number of observed behaviors	645		176		10	
2. The number of times the presence or absence of sniffing was confirmed	558	86.5 (558/645)	164	93.2 (164/176)	7	70.0 (7/10)
2. 1. Only immediately before	512^b^	91.8 (512/558)	84^a^	51.2 (84/164)	4^a^	57.1 (4/7)
2. 2. Only immediately after	0^a^	0.0 (0/558)	8^b^	4.9 (8/164)	0^a,b^	0.0 (0/7)
2. 3. Both before and after	3^a^	0.5 (3/558)	63^b^	38.4 (63/164)	0^a,b^	0.0 (0/7)
2. 4. Without sniffing	43^b^	7.7 (43/558)	9^b^	5.5 (9/164)	3^a^	42.9 (3/7)

^a,b^Denotes a subset of the excretion category (urine spray, scraping, and other excretion) whose ratio was not significantly different from the ratio of the subset of the other category (*p* < 0.05: χ^2^ test).

### Differences based on sex and age class

Urine spraying was observed in 3 out of 5 adult females (average number in adult females in 25 min: 0.81 ± 1.32 times, n = 43), 13 out of 14 adult males (5.33 ± 3.98 times, n = 111), 0 out of 3 sub-adult females (0 times, n = 20), and 0 out of 3 male sub-adults (0 times, n = 34) (Table 2). Scraping was observed in males only (10 out of 14 individuals: 1.59 ± 2.15 times, n = 111), never observed in sub-adults, and not confirmed in females, including adults (0 times, n = 43). The frequencies of urine spraying and scraping did not increase or decrease with age in both sexes. Other excretions were observed in not only adults but also sub-adults regardless of sex (urination: 1 sub-adult male and 1 adult male; defecation: 3 times in 2 sub-adult females, 4 times in 3 adult males, 1 time in 1 adult female).

### Target location for urine spraying and scraping

**Figure 3:**
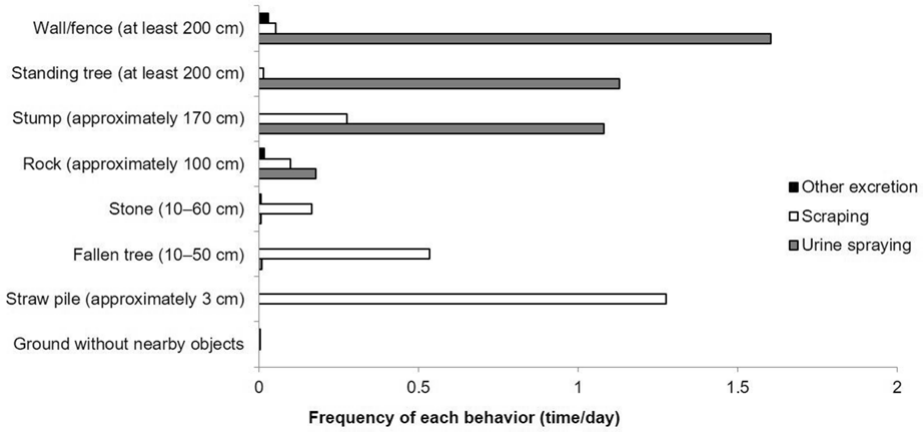
Frequency of urine spraying, scraping, and other excretion at each target location. The daily observation time was 25 min after release.

The locations of urine spraying and scraping were compared (Fig. 3, Table 6). Urine spraying was significantly frequently performed on high objects, like walls or fences (1.60 times/day) and standing trees (1.13 times/day) (χ^2^ test, *p* < 0.05). In contrast, scraping was frequently observed on low-lying objects, straw piles (1.28 times/day), a fallen tree (0.54 times/day), and stones (0.17 times/day) but there were no significant differences from urine spraying and/or other excretion (χ^2^ test, *p* > 0.05). In addition, of the 10 other excretions, 6 were observed on the ground without nearby objects (Online resource 1; Other excretion besides urine spraying and scraping, Supplementary Table 1c).

**Table 6:** Target location for urine spraying and scraping.

	Ground without nearby objects	Straw pile (approximately 3 cm)	Fallen tree (10–50 cm)	Stone (10–60 cm)	Rock (approximately 100 cm)	Stump (approximately 170 cm)	Standing tree (at least 200 cm)	Wall/fence (at least 200 cm)
Total number of observations days^A^	210	29	112	138	112	112	146	210
**Urine spraying**								
Times	0^a^^,b^	0^a^	1^a^	1^a^	20^b,c^	121^c^	165^d^	337^d^
(times/days)	(0)	(0)	(0.01)	(0.01)	(0.18)	(1.08)	(1.13)	(1.6)
**Scraping**								
Times	1^b,c^	37^a^	60^a^	23^a^	11^c^	31^c^	2^b^	11^b^
(times/days)	(0)	(1.28)	(0.54)	(0.17)	(0.1)	(0.28)	(0.01)	(0.05)
**Other excretion**								
Times	6^c^	0^a^^,b^	0^a^^,b^	2^b^	1^a^^,b^	0^a^	0^a^	1^a^
(times/days)	(0.03)	(0)	(0)	(0.01)	(0.01)	(0)	(0)	(0)

^A^The “day” indicate the day individuals were monitored in the presence of the target object. The daily observation time is 25 min after release.

^a,b,c,d^Each superscript represents a subset of the target object category whose ratio was not significantly different from the ratio of the subset of other categories (p < 0.05: χ^2^ test).

### Differences in urine spraying and scraping, depending on the scent condition at the observation location

The mean number of urine spraying events (Table 2) was significantly higher in shared enclosures (6.22 ± 3.71 times, n = 69) than in monopolized enclosures (4.11 ± 4.06 times, n = 44) (Mann–Whitney *U* test, *p* < 0.05) for adult males but not adult females (0.67 ± 1.07 times, n = 15 vs. 0.89 ± 1.42 times, n = 28, respectively) (Mann–Whitney *U* test, *p* = 0.764). Scraping was significantly higher in the shared enclosures (9 out of 10 adult males, 2.54 ± 2.25 times, n = 69) than in the monopolized enclosures (1 out of 4 adult males, 0.02 ± 0.15 times, n = 44) (Mann–Whitney *U* test, *p* < 0.05).

**Figure 4:**
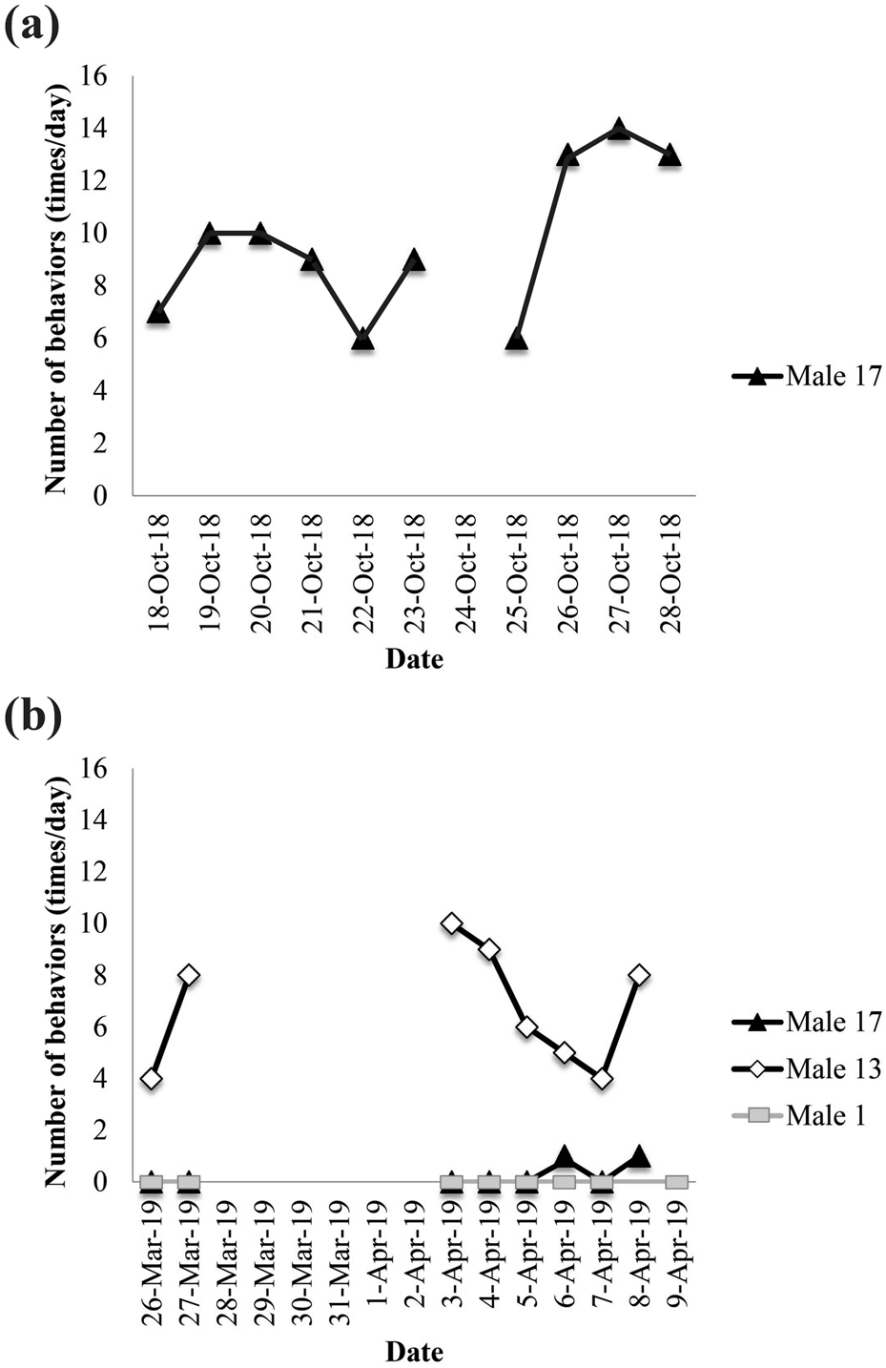
Difference in the number of urine sprays depending on the release method. (**a**) The frequency of urine spraying observed when only Male 17 was released and had exclusive use of the enclosure. (**b**) The frequency of urine spraying when Males 17, 1, and 13 were alternately and sequentially released into the enclosure normally used by Male 13. The daily observation time was 25 min after release.

On the other hand, in the monopolized enclosure, when Male 17 was released alone in an area inhabited by other male individuals (Males 1 and 13), the mean of the number of urine spraying events significantly decreased (0.25 ± 0.43 times, n = 8) from that observed in the monopolized enclosure when it was not inhabited by other males (5.50 ± 5.13 times, n = 18) (Fig. 4) (Mann–Whitney *U* test, *p* < 0.05). The mean numbers of urine spraying events for Males 1 and 13 were 0 (n = 8) and 6.75 ± 2.17 times (n = 8), respectively. Scraping was not observed in any of the three animals (Males 1, 13, and 17).

## Discussion

### Urine spraying and scraping as potential scent-marking

The urine spraying and the scraping were reported in other felids^6,20,21^. In this study, only half of the other excretion instances were accompanied by sniffing, whereas almost all urine spraying and scraping events were accompanied by sniffing, indicating that these are scent-markings. The sniffing was also often observed immediately before urine spraying and scraping. Given the significant association of sniffing before excretion, especially with regard to the scraping, the presence or absence of a scent on the object was thought to be a trigger.

Furthermore, during the scraping, liquid secretions thought to originate from the anal glands, were released. Domestic cats have scent glands in the anal sac^22^. The presence of secretions from the anal sac has also been confirmed in not only tigers, lions *(Panthera leo*), and bobcats (*Lynx rufus*), but also in cheetahs^1,6,23^; however, this study was the first to investigate their role in excretion. Generally, secretions are considered to be caused by health problems or estrus, but in this study, none of the individuals had health problems, and all secretions were observed only in males. Therefore, it was thought that the secretion was produced by the scent glands and contributed to a stronger smell than only urine and feces.

### Variations based on sex

Urine spraying was observed only in adult males and females, and was more frequent in males, as reported in other felids^4–6,9,24^. In wild cheetahs, although urine spraying and scraping have been observed as scent-making, the frequency of scent-marking is known to be substantially higher in territorial than in non-territorial males and in females^15,16,25^, and the marking locations are concentrated in the core area of the male territories^16^. The territories of a single male cheetah or a male group are relatively small and exclusive, whereas the relatively large home ranges of non-territorial males (also known as “floaters”) overlap with each other and with those of females^15,16^. A male’s home range is also larger than that of a female^15,16,26,27^. Male cheetahs rarely encounter other males because they communicate via marking posts^28^. Given these reports, the frequent urine spraying by males may help prevent encounters between males. In addition, observations of captive cheetahs have shown a significantly positive correlation between urinary spraying frequency and fecal estradiol content in female cheetahs^19^. Therefore, as Cornhill and Kerley^24^ mentioned, female urine spraying is caused by estrus, and male urine spraying is intended as a home range marker for other males or as a sign for females.

The action of scraping using the hind paws has been reported to occur in both males and females in servals, lions, tigers, black-footed cats, etc.^2,5–7,29^; however, this behavior was only observed in adult males in this study. Sunquist and Sunquist^3^ reported that female cheetahs also perform the scraping. In this study, we only recorded observations when the cheetahs were released in the outdoor enclosures, and not when they were in the indoor facilities. In 43.6% of the scraping events, the males excreted feces. During the observation period, the females defecated in the indoor facilities, and no defecation was observed in the outdoor enclosures. It is possible that no scraping action was observed among the females because defecation was not observed in the outdoor enclosure. In indoor facilities, the cheetahs were in a completely monopolized enclosure; hence, the females defecated in their own spaces. There was a difference in the defecation sites and frequency of scraping between the males and females; this was attributed to the sex difference in scent-marking.

### Differences in target height for each behavior

Urine spraying was frequently done on objects approximately 170 cm or higher, such as walls or fences, standing trees, and stumps, whereas scraping was observed on low-lying objects on the ground, such as a straw pile approximately 3 cm high and a fallen tree that was 10–50 cm high. In other words, the cheetah engaged in urine spraying and scraping depending on the object nearby. This might indicate the functional role of these behaviors. This is consistent with previous findings of urine spraying by tigers being more frequent in wooded forests than in grasslands, with few prominent objects, and scraping being more common in the latter^6^. In addition, in a study that investigated the place where the smell of the urine of domestic cats is likely to remain, the smell persisted for a long time on rough surfaces, areas covered with moss, and overhanging slopes^30^. Even for cheetahs living in the savanna woodlands, where there are comparatively fewer upright objects than in the habitat of felids living in the forest, increasing the chances of transmitting information via not only urine spraying but also by the scraping might be more important. On the other hand, in their natural habitat, there are some large carnivores like lions and leopards (*Panthera pardus*). Wild cheetahs tend not to visit the sites where such carnivores’ scent-mark is present^31^, suggesting that they might confine their marking to specific sites devoid of other carnivores’ scent. Further research is needed to determine how wild cheetahs use urine spraying and scraping. In this study, scraping was frequently observed even on tall stumps and rocks if they were within the cheetahs’ reach. Scraping by wild cheetahs has been also observed on trees^32^. Zoos other than Zoo C had few prominent horizontal objects. Therefore, the presence of straw piles, fallen trees, stumps, and rocks may have elicited the scraping.

### Differences in housing conditions

In zoos C and D, where animals shared enclosures, the frequency of both urine spraying and scraping by males was higher than in the males in the monopolized enclosures. They possibly showed a more frequent scent-marking to strengthen their home range claims when sharing the exhibition space^15^. Regarding the scraping, Zoo C had at least four low and horizontal objects (straw piles, fallen tree, stones, and rocks), and scraping was frequently observed. As mentioned above, the placement of objects might have elicited the scraping.

In this study, the frequency of urine spraying decreased when the submissive individual (Male 17) was released in the enclosure where the dominant individual (Male 13) was previously released. Among wild cheetahs, territorial males have been reported to mark their territories more often than non-territorial males^17,25^. Therefore, the difference in the number of markings is considered to be related to whether or not the target individual is within the territory, and it is highly possible that the dominant/submissive relationship between males at that location has an effect on marking.

### Function of scraping using hind paws

Other felid studies have reported scraping in tigers, pumas, jaguars, clouded leopards, and small felids^6,10,20,21,32,33^; however, there are fewer studies on different types of scraping. In certain species, such as jaguars and pumas, scraping using hind paws is more frequent than urine spraying^33^. From this study, the use of secretions was confirmed in the scraping, and it was considered to be a significant marking of the cheetah.

The possible functions of scraping include: (1) dispersing the smell of excrement, (2) placing the smell of excrement on the hindlegs, (3) smearing the objects with excrement, and (4) adding the scent of the hind paws. Domestic cats are known to cover their feces with soil^34^; however, in this study, the cheetahs did not cover the feces with soil and were not observed to scrap only after excretion. Therefore, scraping using hind paws was not meant for concealing urine and/or feces. The results of this study suggest that the scrapings were mostly performed during and after excretion for any of the aforementioned functions. However, 43.2% of the observed scraping events were performed before excretion, and in these cases, the functions 1–3 did not apply, since we did not observe the feces being crushed by scraping the hind paws. As for function 4, domestic cats have sweat glands on the soles of their feet that are thought to retain their smell^35^. Therefore, the sweat glands on the soles of the feet of the cheetahs possible retain the smell of the hind paws as well. Schaller^36^ reported that among tigers, scraping on the grassland was exhibited by scratches in the grass and exposure of the ground, creating a visual effect. In the case of cheetahs, scraping may have the function of creating grooves and ridges on the ground to enhance the visual effect; however, the formation of grooves and ridges were not observed in this study. In certain cases, they scraped against trees and stones. Because trees and stones are not easily deformed, it is hard to say whether the visual effect was enhanced by scraping with their hind paws.

Scraping has been reported in other felids; however, the movement of the hindlimbs is not uniform. For example, in the case of bobcats, behaviors such as kicking back on the ground with no surrounding objects and scattering of soil have been observed during scrapings^20^. The snow leopard slowly moves its hindlimbs on the ground near the rocks, exposing the ground; in fact, Schaller^29^ observed a tiger scraping its hind paws to create a pile of soil [^37^; Kinoshita, personal communication: Online Resource 3; Scraping of snow leopard]. The movement of urine spraying also varies among species. For example, bobcats sometimes squat and urinate on the ground^20^, and snow leopards rub their cheeks against the target object and then spray urine^9^, but cheetahs do not rub their face before urine spraying. Hence, even in the same behavior of “spraying/scraping,” the actions differ. Because felids are widely distributed in various environments, such differences in movements are possibly related to differences in habitat and behavioral functions.

In conclusion, urine spraying and scraping using hind paws were considered scent-markings because they were more strongly associated with sniffing than other excretion. Both behaviors were also observed only in adults; however, urine spraying was confirmed in both sexes and was more frequent in males than in females, whereas scraping was observed only in males. Also, the frequencies of both behaviors were significantly higher in males kept in shared enclosures containing other individuals than in males kept in monopolized enclosures, while there was no difference in the frequencies among females. Hence, there were sex differences in these scent-markings possibly because of the difference in the sociality between the sexes even in captivity; the frequency of scent-markings was affected by the living environment including the number of target objects; urine spraying was frequently done on tall objects such as walls or fences, whereas scraping was more commonly done on low-lying objects near the ground, such as straw piles. To our knowledge, this study is the first to confirm that during the scraping a liquid other than feces and urine was secreted, presumably from the anal glands. Taken together, the results can serve to enhance our knowledge regarding the behavior of cheetahs, help improve management of these animals in captivity as well as breeding and animal welfare ex situ conservation, and help elucidate the kind of habitat that should be preserved for the in situ conservation of cheetahs.

## Supplementary Information

Supplementary Video 1.

Supplementary Video 2.

Supplementary Video 3.

Supplementary Information 1.

Supplementary Information 2.

## Data Availability

All data generated or analyzed during this study are included in this published article and its supplementary information files.

## References

[1] Asa CS (1993). Relative contributions of urine and anal-sac secretions in scent marks of large felids. Am. Zool..

[2] Mellen JD (1993). A comparative-analysis of scent-marking, social and reproductive-behavior in 20 species of small cats (Felis). Am. Zool..

[3] Sunquist, M. & Sunquist, F. Olfactory communication in felids. In *Wild Cats of the World* (University of Chicago Press, 2002).

[4] Sunquist, M. & Sunquist, F. Vocal communication in felids. In *Wild Cats of the World.* 421–424 (University of Chicago Press, 2002).

[5] Molteno AJ, Sliwa A, Richardson PRK (1998). The role of scent marking in a free-ranging, female black-footed cat (*Felis nigripes*). J. Zool..

[6] Smith JLD, Mcdougal C, Miquelle D (1989). Scent marking in free-ranging tigers, *Panthera Tigris*. Anim. Behav..

[7] Geertsema AA (1985). Aspects of the ecology of the serval Leptailurus serval in the Ngorongoro Crater, Tanzania. Neth. J. Zool..

[8] Feldman HN (1994). Methods of scent marking in the domestic cat. Can. J. Zool..

[9] Freeman H (1983). Behavior in adult pairs of captive snow leopards (*Panthera uncia*). Zoo. Biol..

[10] Allen ML, Wittmer HU, Wilmers CC (2014). Puma communication behaviours: Understanding functional use and variation among sex and age classes. Behaviour.

[11] Johnson RP (1973). Scent marking in mammals. Anim. Behav..

[12] Marnewick KA, du Bothma JP, Verdoorn GH (2006). Using camera-trapping to investigate the use of a tree as a scent-marking post by cheetahs in the Thabazimbi district. S. Afr. J. Wildl. Res..

[13] Durant, S., Mitchell, N., Ipavec, A. & Groom, R. *Acinonyx jubatus.*10.2305/IUCN.UK.2015-4.RLTS.T219A50649567.en (2015).

[14] Frame, G. W. Cheetah social organization in the Serengeti ecosystem, Tanzania. In *Paper Presented at the Animal Behavior Society, Colorado* (1980).

[15] Caro, T. M. *Cheetahs of the Serengeti Plains: Group Living in an Asocial Species* (University of Chicago Press, 1994).

[16] Melzheimer J, Heinrich SK, Wasiolka B, Mueller R, Thalwitzer S, Palmegiani I, Weigold A, Portas R, Roeder R, Krofel M, Hofer H, Wachter B (2020). Communication hubs of an asocial cat are the source of a human–carnivore conflict and key to its solution. PNAS.

[17] Caro TM, Collins DA (1987). Ecological characteristics of territories of male cheetahs (*Acinonyx Jubatus*). J. Zool..

[18] Eaton, R. L. (1974) The cheetah. In *The Cheetah—The Biology, Ecology, and Behavior of An Endangered Species* 16–40 (Van Nostrand Reinhold Company, 1974).

[19] Wielebnowski N, Brown JL (1998). Behavioral correlates of physiological estrus in cheetahs. Zoo. Biol..

[20] Allen ML, Wallace CF, Wilmers CC (2015). Patterns in bobcat (*Lynx rufus*) scent marking and communication behaviors. J. Ethol..

[21] Allen ML (2015). The role of scent marking in mate selection by female pumas (*Puma concolor*). PLoS One.

[22] McColl L (1967). The comparative anatomy and pathology of anal glands. Arris and Gale lecture delivered at the Royal College of Surgeons of England on 25th February 1965. Ann. R. Coll. Surg. Engl..

[23] Hashimoto Y, Eguchi Y, Arakawa A (1963). Histological observation of the anal sac and its glands of a tiger. Nihon Juigaku Zasshi Sci..

[24] Sunquist ME (1981). The social organization of tigers (*Panthera tigris*) in Royal Chitawan National Park, Nepal. Smithson Contr. Zool..

[25] Cornhill KL, Kerley GI (2020). Cheetah behaviour at scent-marking sites indicates differential use by sex and social rank. Ethology.

[26] Houser AM, Somers MJ, Boast LK (2009). Home range use of free-ranging cheetah on farm and conservation land in Botswana. S. Afr. J. Wildl. Res..

[27] Marnewick K, Somers MJ (2015). Home ranges of cheetahs (*Acinonyx jubatus*) outside protected areas in South Africa. Afr. J. Wildl. Res..

[28] Broekhuis F, Madsen EK, Keiwua K, Mcdonald DW (2019). Using GPS collars to investigate the frequency and behavioural outcomes of intraspecific interactions among carnivores: A case study of male cheetahs in the Maasai Mara, Kenya. PLoS One.

[29] Schaller, G. B. *The Serengeti Lion*. (University of Chicago Press, 1972).

[30] Mohorovi M, Krofel M (2020). The scent world of cats: Where to place a urine scent mark to increase signal persistence. Anim. Biol..

[31] Cornhill KL, Kerley GI (2020). Cheetah communication at scent-marking sites can be inhibited or delayed by predators. Behav. Ecol. Sociobiol..

[32] Allen ML, Wittmer HU, Setiawan E, Jaffe S, Marshall AJ (2016). Scent marking in Sunda clouded leopards (*Neofelis diardi*): Novel observations close a key gap in understanding felid communication behaviours. Sci. Rep..

[33] Harmsen BJ, Foster RJ, Gutierrez SM, Marin SY, Doncaster CP (2010). Scrape-marking behavior of jaguars (*Panthera onca*) and pumas (*Puma concolor*). J. Mammal..

[34] Wemmer, C. & Scow, K. How animals communicate. In *Communication in the Felidae with Emphasis on Scent Marking and Contact Patterns* (ed. Seboek, T.) 749–766 (Indiana University Press, 1977).

[35] Meyer W, Bartels T (1989). Histochemical study on the eccrine glands in the foot pad of the cat. Basic Appl. Histochem..

[36] Schaller, G. B. *The Deer and the Tiger* (University of Chicago Press, 1967).

[37] Kinoshita K (2009). Relationship between sexual behaviors and fecal estrogen levels in a female snow leopard (*Uncia uncia*) and a female cheetah (*Acinonyx jubatus*) under captivity. Jpn. J. Zoo Wildl. Med..

