# Supplementary material for: Characteristics of urine spraying and scraping the ground with hind paws as scent-marking of captive cheetahs (Acinonyx jubatus)
Source: Sci Rep. 2022 Sep 16;12:15594. doi: 10.1038/s41598-022-19257-7 (PMC9481607; doi:10.1038/s41598-022-19257-7)
Supplement: Supplementary file 4 — Supplementary Information 1. [file 41598_2022_19257_MOESM4_ESM.docx]

**Supplementary information**

Supplementary table:

Raw data on the number of urine spraying (a), scraping (b), and other excretion (c)

Online Resource 1:

Other excretion besides urine spraying and scraping

Online Resource 2:

Posture when excreting secretion during scraping behavior

Online Resource 3:

Scraping of snow leopard
